# Deep Brain Stimulation for Cerebellar Ataxia: A Systematic Review on Indications, Targets and Outcomes

**DOI:** 10.1007/s12311-026-01999-z

**Published:** 2026-04-21

**Authors:** G Mantovani, P Antenucci, E Collautti, A Gozzi, M Sensi, MA Cavallo, A Scerrati

**Affiliations:** 1 Department of Translational medicine, University of Ferrara, Ferrara, Italy; 2 Neurosurgery Unit, Sant’Anna University-Hospital, Ferrara, Italy; 3 Neurology Unit, Department of Neuroscience, Sant’Anna University Hospital, Ferrara, Italy; 4 University of Ferrara, Ferrara, Italy; 5 Sant’Anna University Hospital, Via A Moro 8, Ferrara, 44124 Italy

**Keywords:** Cerebellum, Deep brain stimulation, Ataxia, Systematic review

## Abstract

Background. Cerebellar ataxias are a heterogeneous group of disorders characterized by progressive impairment of coordination, gait and balance, for which effective symptomatic treatments remain limited. Abnormal activity within the deep cerebellar nuclei, secondary to cerebellar cortical degeneration, represents a key pathophysiological mechanism. Deep Brain Stimulation has therefore emerged as a potential therapeutic strategy to modulate cerebellar output pathways. Objective. To systematically review the available clinical and preclinical evidence on the efficacy, safety, and stimulation targets of Deep Brain Stimulation for cerebellar ataxia. Methods. A systematic search of MEDLINE (PubMed) and Scopus was conducted in accordance with PRISMA 2020 guidelines. Studies involving human subjects with cerebellar ataxia treated with Deep Brain Stimulation were included, alongside relevant preclinical animal studies. Data on ataxia aetiology, stimulation targets, clinical outcomes and adverse effects were extracted and qualitatively analyzed. Results. Fifteen clinical studies (27 patients) and seven preclinical studies were included. The Dentate Nucleus was the most frequently targeted structure in human studies and was associated with modest but clinically meaningful improvements in ataxic symptoms, particularly in selected hereditary and post-lesional forms. Tremor-dominant phenotypes benefited consistently from thalamic stimulation. Preclinical studies highlighted the Interposed Nucleus as a promising novel target, demonstrating robust restoration of motor coordination and cerebello-thalamo-cortical signaling in animal models. Conclusions. Deep Brain Stimulation appears to be a feasible and potentially effective therapeutic option for selected patients with cerebellar ataxia. While current clinical evidence remains limited and heterogeneous, converging data suggest that modulation of cerebellar nuclei—particularly the Dentate Nucleus and the Interposed Nucleus—represents a promising avenue for future translational research.

## Introduction

Cerebellar ataxia comprises a heterogeneous group of disorders characterized by impairment of the motor control pathways subserving cerebellar function. Their aetiology is diverse, encompassing acquired forms of infectious, immune-mediated, traumatic, ischemic, or toxic origin, as well as neurodegenerative and genetically determined conditions. Among hereditary forms, the most relevant include autosomal recessive conditions such as Friedreich’s ataxia and ataxia-telangiectasia, and autosomal dominant entities such as the spinocerebellar ataxias (SCAs) and the episodic ataxias [1].

Epidemiologically, the overall prevalence of hereditary cerebellar ataxia is estimated to range from 2.7 to 3.3 cases per 100,000 inhabitants, though this likely underestimates the true incidence due to diagnostic variability and underreporting [2]. Among inherited forms, Friedreich’s ataxia represents the most common type in Europe, with regional prevalence ranging from 1 in 20,000 to 1 in 250,000 [3]. SCAs collectively affect approximately 1–3 individuals per 100,000, showing marked geographical variation [4]. The prevalence of acquired ataxias remains difficult to quantify due to the heterogeneity of underlying causes.

Therapeutic strategies for cerebellar ataxia depend on the underlying aetiology, and when possible, treatment is directed at the primary cause; however, for most patients, management remains symptomatic, and pharmacological interventions aim to reduce tremor, spasticity, or episodic attacks [5–7] and rehabilitative and supportive therapies intended to preserve functional independence and quality of life provide only limited benefits [8], particularly in hereditary-degenerative forms. Recently, disease-modifying agents such as omaveloxolone, approved for Friedreich’s ataxia in 2023, have shown clinical benefits by improving neurological function and slowing disease progression [9, 10].

Despite these therapeutic advances, the prognosis of ataxia remains poor. Non-invasive cerebellar stimulation (NICS) has emerged as an important therapeutic resource in recent years [11]. It is well established that abnormal excitability within the deep cerebellar nuclei (DCN), secondary to cerebellar cortical degeneration, represents a key pathophysiological mechanism underlying ataxic symptoms [12–15]. In particular, dysregulation of the cerebello-thalamo-cortical and cerebello-spinal circuits hampers predictive motor signalling, resulting in the uncoordinated and inaccurate motor outputs responsible for imbalance, gait disturbances, and the typical motor symptomatology of these disorders [16, 17]. Modulation of neural activity aimed at resynchronizing abnormal networks that converge on common targets—most notably the cerebellar cortex, the DCN, and subcortical deep brain nuclei—provides a rationale for the promising results obtained using specific NICS protocols, particularly transcranial magnetic stimulation (TMS) and transcranial direct current stimulation (ctDCS) [11]. However, the lack of medium- to long-term benefits, the strong dependence of improvement on the number and duration of stimulation sessions, and the limited ability to target specific structures make NICS a constrained therapeutic option.

Deep Brain Stimulation (DBS), a well-established neurosurgical technique used for Parkinson’s disease, essential tremor, dystonia, and obsessive–compulsive disorder, has been shown to effectively alleviate motor symptoms in various movement disorders by modulating pathological neuronal activity. This technique represents a potential therapeutic option for the treatment of cerebellar ataxias, particularly by overcoming the practical limitations associated with NICS [18].

Given the growing number of reports exploring DBS for cerebellar ataxia, a systematic evaluation of its efficacy, safety, and clinical applicability is warranted. Therefore, the aim of this systematic review is to assess the effectiveness of Deep Brain Stimulation in the treatment of ataxic symptoms of cerebellar origins, summarizing current evidence about target nuclei and identifying key knowledge gaps to inform future research.

## Materials and Methods

This systematic review was conducted in accordance with the PRISMA 2020 guidelines. The review protocol was registered in PROSPERO (registration n° 1051214).

The primary objective was to evaluate the efficacy and safety of DBS in the treatment of ataxia, with particular attention to the targeted brain regions, clinical outcomes and potential adverse effects. Where available, data about stimulation parameters were also collected.

A comprehensive literature search was performed across the databases MEDLINE (Pubmed) and Scopus. The search strategy included combinations of the following keywords and MeSH terms: “ataxia”, “deep brain stimulation” and “DBS”. Boolean operators AND/OR were used to refine the search. The search was limited to articles published in English up to April 2025. Additional studies were identified by manually screening the references of relevant articles.

Eligibility criteria included clinical studies involving human subjects diagnosed with any form of cerebellar ataxia and treated with DBS. Both observational studies and interventional trials (RCT and non-randomized) were considered. Case reports and case series were also included. Pre-clinical models and animal studies were included but separately analyzed for pathophysiological insights.

Two reviewers (GM and EC) independently screened titles and abstracts, followed by full-text assessments for eligibility. Data were extracted using a standardized form and included different aetiologies of cerebellar ataxia, DBS target, main clinical symptoms, outcome measures (such as improvement in motor function, coordination and quality of life), follow-up duration and reported complications.

Since the focus of this review is the DBS for the treatment of ataxic symptoms in cerebellar ataxias, studies that used DBS in these disorders for the management of other symptoms (dystonia, parkinsonism, myoclonus) were excluded.

For bias assessment of the included studies, the Newcastle–Ottawa Scale (NOS) was considered for non-randomized observational studies. The Cochrane Risk of Bias 2.0 (RoB 2) tool was employed for RCTs.

## Results

A total of 567 records were identified through database searching, with 202 records from PubMed and 365 from Scopus.

After removing 156 duplicate records, 411 records remained for title and abstract screening. Of these, 339 were excluded based on the predefined eligibility criteria. A total of 72 full-text articles were sought for retrieval.

Of the retrieved full-text articles, 26 were assessed for eligibility. Among these, 11 were excluded because they did not focus on the condition of interest and their outcomes were not relevant to the objectives of the review.

Ultimately, 15 studies met the inclusion criteria and were included in the final review.

### Study Characteristics 

Table 1 provides a comprehensive overview of the included studies, highlighting substantial heterogeneity in terms of aetiologies, DBS targets, main symptoms and clinical outcomes.

The total number of participants across all human studies was 27 (Diniz et al. (2021) [19] include 2 patients from Texeira (2015) [20]and Cury (2019) [21])

The included studies consisted of 10 case reports, 4 case series, 1 RCTs and 7 preclinical animal studies.

Table 2 provides an overview of the preclinical included studies applying DBS in animal model of ataxia.

#### Ataxia Aetiologies and Patient Populations

The included studies represent a wide range of ataxia aetiologies, including autosomal dominant forms such as Spinocerebellar Ataxias (SCA1, SCA2, SCA3, SCA3-PS, SCA12, SCA17, SCA27A), autosomal recessive conditions like Friedreich’s Ataxia, mitochondrial ataxias (e.g., MERRF) [12] and acquired forms including post-stroke and trauma-related ataxias.

FXTAS (Fragile X-associated Tremor/Ataxia Syndrome) was frequently included because of its significant tremor component [13–15, 22, 23].

Several animal studies using genetic models or cerebellar lesions were also qualitatively analyzed to better understand how DBS works and to evaluate its effects before clinical application [24–30].

#### Targeted Brain Structures

Across all studies, a total of 7 distinct DBS targets were reported, including the dentate nucleus (DN), thalamic targets such as the ventral intermediate nucleus (VIM) and ventral oral nucleus (Vo/VoP), the posterior subthalamic area (PSA), the zona incerta (ZI), the subthalamic nucleus (STN) and globus pallidus internus (GPi).

The DN was the most common stimulated structure, especially in hereditary and post-lesional cerebellar ataxias and in cases where the ataxic component was predominant, reflecting its critical role in cerebello-thalamo-cortical connectivity.

Finally, some pre-clinical studies targeted the Interposed Nucleus (IN) and fastigial nucleus to explore modulation of cerebellar output in animal models of ataxia [24, 25, 29, 30].

#### Clinical Outcomes

Outcomes on Ataxia were most frequently quantified using standardized scales such as the Scale for the Assessment and Rating of Ataxia (SARA), and in some cases with the International Cooperative Ataxia Rating Scale (ICARS).

Most studies reported significant improvements in tremor [13–15, 19–23, 31–34] and dystonia [35] while improvements in ataxic core features such as gait, stance and coordination were more variable.

Selected studies targeting the DN showed inconstant but clinically meaningful improvement in SARA scores, particularly in patients with post-lesional or SCA3-related ataxia [12, 14, 15, 19–21, 31, 32, 35]. These improvements typically ranged from 20% to 35% over 3 to 12 months of follow-up. However, isolated cases reported greater reductions - exceeding 40% - in the short term [15, 32, 35]. Conversely, other studies observed no benefit on coordination despite good tremor control [14, 22, 23], underscoring the variability in response and the symptom-specific nature of DBS efficacy.

Only two papers reported complete SARA subscore [12, 35]. Considering axial symptoms Kumar et al. [12] found no benefit on gait, stance and sitting, while observing an improvement of 66% in speech. Differently, Cui [35] reported an improvement of 43% in gait, 50% in stance, 50% in sitting and 25% in speech.

In general, adverse effects were mild or reversible, with a few cases of stimulation-induced dysarthria or worsened ataxia in cases treated for tremor-dominant symptoms being successfully mitigated through reprogramming [23] or mild transient numbness [34].

**Fig. 1:**
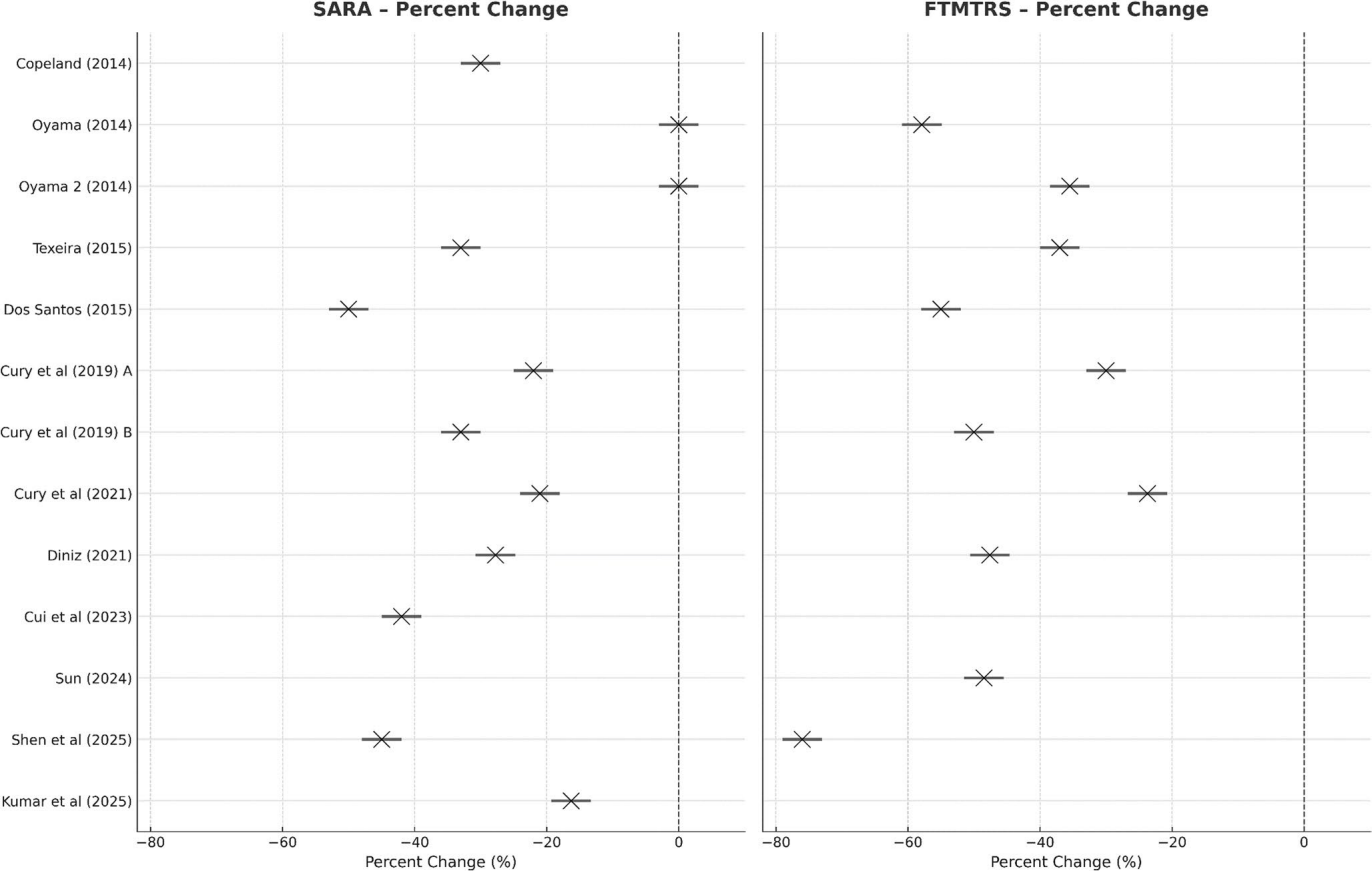
Percent Changes in studies reporting SARA and FTMTRS

All studies reporting clinical outcomes with SARA and FTMTRS were separately analyzed. Although studies heterogeneity prevented from a formal meta-analyses (9 case reports, 1 RCT and 1 case series), mean SARA and FTMTRS variations in the post-operative period were plotted in percentage. The result is showed in Fig. 1.

**Table 1:** Study characteristics

*N*	Study	Type of study	Number of patients – follow up (months)	Type of ataxia	DBS target and stimulation parameters (median ± IQR)	Major symptoms (clinical target)	Quantified outcomes	
1	Cury et al. (2022) [31]	Randomized double-blind crossover pilot trial	5 - 6	SCA3 and post-lesional ataxia	DN 1.6 **±** 1.05 mA 110.5 **±** 91 µs 19 **±** 51 Hz	Tremor Ataxia	SARA: mean decrease by 21.02% (*p* = 0.223); FTMTRS: mean decrease 23.74% (*p* = 0.039); PGIC (Patient Global Impression of Change): 6 (*p* = 0.038)	
2	Diniz et al. (2021) [19]	Case series (including patients from Texeira (2015) and Cury (2019))	3 - NA	post-stroke and SCA-3 ataxia	DN: refined by DRTT tractography	Tremor Ataxia	SARA: mean decrease by 27.8% FTMTRS: mean decrease by 47.6%	
3	Oyama et al. (2014) [22]	Case series	5 - 12	SCA2, SCA17, FXTAS, SETX, Idiopathic Ataxia (NOS)	- SCA2: VIM 1.8 V; 90µs; 135 Hz - FXTAS: VIM/VO 3.3 ± 0 V; 135±30µs; 135 ± 0 Hz - Ataxia NOS: VIM 180 V; 135µs; 6 Hz - SCA17/SETX: GPi 3.4 ± 0.9 V; 315±285µs; 142.5 ± 85 Hz	Tremor	SCA2: FTMTRS decrease 49% (1 year) FXTAS: FTMTRS decrease 25% (1 year) Ataxia NOS: FTMTRS decrease 32.5% (6 months) no benefit for ataxia; gait worsened	
4	Weiss et al. (2015) [13]	Case series	3 - 24	FXTAS	Bilateral VIM 4.5 ± 3.2 V; 120 ± 60 µs; 150 ± 45 Hz	Tremor	Tremor reduction sustained; gait regularized; hand function benefit lost after 1–2 years	
5	Kumar et al. (2024) [12]	Case report	1 - NA	MERRF syndrome (mitochondrial ataxia)	Bilateral cZI 1 mA; 60 µs; 30 Hz (best results)	Ataxia	SARA: decrease by 16.3%; Particularly: Ataxic speech improved at 30 Hz; no benefit on stance or gait subcomponent	
6	Shen (2025) [32]	Case report	1 - 12	SCA12	Bilateral PSA 4.23 ± 0.83 V; 60 ± 30µs; 160 Hz	Tremor Ataxia	SARA: decrease by 45% FTMTRS: decrease by 76%	
7	Keller Sarmiento et al. (2025) [33]	Case series	3 - NA	SCA27A	STN	Tremor Imbalance	Two patients SCA27A: significant tremor reduction. One patient also showed balance improvement with 4-AP	
8	Cui et al. (2023) [35]	Case report	1 - 0,5	SCA3/MJD (Machado-Joseph Disease)	Combined Bilateral DN and GPi GPi: 3 V; 90µs; 185 Hz DN: 2 V; 200 µs; 60 Hz	Dystonia Ataxia	SARA: decrease by 42% Burke-Fahn-Marsden Dystonia Rating Scale (BFMDRS) movement: improvement by 30% BFMDRS disability: improvement by 12.5%	
9	Cury et al. (2019) [36]	Case report	1 - 6	SCA3	Bilateral DN 1.9 ± 0.2 V; 182 µs, 16 Hz	Tremor Ataxia	SARA: decrease by 22% FTMTRS: decrease by 30%	
10	Cury et al. (2019) [21]	Case report	1 - 48	Cerebellar ataxia after stroke	Left DN 1.9 mA, 60 µs, 20 Hz	Tremor Ataxia	SARA: decrease by 33%, FTMTRS: decrease by 50%; effect sustained at 4 years	
11	Sun et al. (2024) [34]	Case report	1 - 12	SCA12	PSA e VIM 3.2 ± 0.4 V; 75 ± 30µs; 175 ± 10 Hz	Tremor	FTMTRS: decreased by 48,5% (33,3% improvement in TETRAS) after 1 year of follow-up. Improvement also in quality of life, but cerebellar ataxia remained stable. Numbness was the only mild and transient side effect	
12	Dos Santos Ghilardi et al. (2015) [15]	Case report	1 - 30	FXTAS (Fragile X-associated tremor/ataxia syndrome)	Bilateral VoP/Zona Incerta 3.125 ± 0.25 mA; 91 µs; 176 Hz	Tremor Ataxia	SARA: decreased by 50% FTMTRS: decreased by 55% at 30 months	
13	Oyama et al. (2014) [14]	Case report	1 - 8	FXTAS	Left PSA 2.8 V, 120 µsec, 160 Hz	Tremor	SARA: not changed FTMTRS: decrease by 57,9%. There was an improvement in both ipsilateral (47.3%) and contralateral (68.4%) tremor.	
14	Mehanna et Itin (2014) [23]	Case report	1 - NA	FXTAS	VIM	Tremor	Good tremor control. Ataxia worsened after second (bilateral) DBS;	
15	Teixeira (2015) [20]	Case report	1 - 12	Cerebellar ataxia after stroke	Left DN 1.4 mA; 2.8 V; 60 ms; 20 Hz	Tremor Ataxia	SARA: decrease by 33%. FTMTRS: decrease by 37%. PGIC: 6	

**Table 2:** Preclinical studies using animal models

Study	Type of ataxia	DBS target (name, coordinates)	Proposed mechanism	Quantified outcomes		
Kumar et al. (2022) [24]		Cerebellar ataxia (Lhx1/Lhx5 Purkinje cell knockout mice)	IN-DCN in the right hemisphere at the following coordinates relative to bregma: anteroposterior (AP) = − 6.4 mm, mediolateral (ML) = − 1.3 mm, and dorsoventral (DV) = + 2.5–3 mm from the skull surface	Stimulation based on EMG to restore cerebello-thalamo-cortical signaling	The optimal IN-DCN DBS parameter reversed motor deficits in ataxia mice as detected by animal behavioral tests and EMG recording. Anti-inflammatory cytokines (IL-13, IL-4) were upregulated after IN-DCN DBS, which play key roles in neural excitability	
Kumar et al. (2024) [25]		Cerebellar ataxia (Lhx1/Lhx5 Purkinje cell knockout mice)	IN-DCN, in the right hemisphere at the following coordinates relative to bregma: anteroposterior (AP) = − 6.4 mm; mediolateral (ML) = − 1.3 mm; dorsoventral (DV) = + 2.5–3 mm from the skull surface	Closed-loop stimulation based on EMG to restore cerebello-thalamo-cortical signaling	Full restoration of motor activity, EEG and neurospike features in mice; closed-loop more effective than open-loop	
Miterko et al. (2019) [26]		Stroke, dystonia, epilepsy (rodent models); cerebellar ataxia (preclinical)	DN, IN, centrolateral nucleus of the thalamus; coordinates vary by model	Enhancing excitability of dentato-thalamo-cortical pathway; restoring cerebellar output; suppressing seizure-like activity	Motor improvement in dystonia and stroke models; seizure suppression via optogenetic stimulation of DCN	
Kumar e Ma (2023) [27]		Cerebellar ataxia in a mouse model (pcd5J mice)	DCN	Cerebello-thalamo-cortical computational neural network model to simulate a mouse model of cerebellar ataxia (pcd5J mice) by manipulating cerebellar bursts through reduction of GABAergic inhibitory input	Ataxia mice showed normalization of the motor cortex LFP (Local field potentials)	
Anderson et al. (2019) [28]		Degenerative Cerebellar Ataxia (shaker rat model)	Dorsal DN: 3.5 mm lateral, 11 mm posterior, 5.9 mm ventral from bregma	Low-frequency (30 Hz) stimulation improves dentato-thalamocortical output	Improved gait, reduced tremor and falls in rat model	
Miterko et al. (2021) [29]		Hereditary ataxia (Car8 mouse model)	IN-DCN: coordinates not specified	DBS at 13 Hz normalizes locomotor EMG and improves coordination	Restored gait and coordination in mice; best effects with early DBS + skilled exercise	
Gruver et al. (2024) [30]		Mouse model of cerebellar dysfunction (basic science)	CN neurons in the fastigial nucleus with optogenetic stimulation	Purkinje-CN synaptic connectivity across functional zones shapes CN output and integration	Structured Purkinje-CN connectivity observed; implications for targeted neuromodulation	

Overall, the risk of bias was considered high across most studies due to small sample sizes, lack of standardized protocols and frequent reliance on subjective outcome reporting.

Cury et al. (2021) [31] was rated as having “some concerns,” mainly due to unclear randomization and potential selective reporting. The evaluation is summarized in Table 3.

**Table 3:** Risk of bias (RoB 2.0) evaluation table for RCT

Study	Randomization process	Deviations from intended interventions	Missing outcome data	Measurement of outcome	Selection of reported results	Overall risk of bias
Cury et al. (2021) [31]	Some concerns	Low risk	Low risk	Low risk	Some concerns	Some concerns

## Discussion

This systematic review analyzed the existing evidence on the use of Deep Brain Stimulation (DBS) in the treatment of cerebellar ataxias, a group of heterogeneous neurodegenerative and acquired disorders characterized by impaired coordination and balance. Given the lack of curative treatments, DBS has been explored as a symptomatic intervention, targeting the most disabling motor symptoms, primarily gait instability and incoordination. The literature reflects an increasing interest in neuromodulation for ataxia, but, at the same time, the findings reveal a heterogeneous and evolving landscape, characterized by variable clinical responses, a wide range of DBS targets and a lack of standardized protocols.

### Clinical Outcome Measures and Evaluation Methods

In terms of outcome assessment, only a subset of studies employed validated scales for quantifying motor improvement. The SARA scale was the primary tool used to measure changes in core ataxia symptoms in studies targeting the DN [12, 14, 15, 19–21, 31, 34, 35]. For tremor-dominant cases, especially in FXTAS, FTMTRS [14, 15, 19–22, 31, 32, 34, 36] and TETRAS [34] were more frequently used.

However, several studies relied on subjective impressions or semi-quantitative clinical observations rather than validated rating scales, which substantially limits the reproducibility, comparability and objectivity of the reported outcomes. In these cases, improvement was often described in vague terms without standardized measures to quantify the extent or consistency of the effect. This lack of rigorous assessment undermines the strength of the evidence and complicates cross-study comparisons.

Long-term durability of DBS effects remains uncertain. As summarized in Table 1, follow-up durations are short and inconsistent across studies, with only a few cases monitored beyond two years. This limited evidence does not allow firm conclusions on whether clinical benefits are sustained or gradually attenuate with disease progression.

### Preclinical Evidence and Animal Targets

The cerebellar nuclei constitute key computational hubs that integrate cerebellar cortical output with extracerebellar inputs to shape motor control and motor learning. Classical models posit that mossy fibers collateralize uniformly to both cerebellar cortex and nuclei, establishing a canonical circuit in which direct excitation of nuclear neurons is balanced by Purkinje cell–mediated feedforward inhibition, thereby enabling forward-model computations that anticipate the slower sensory feedback [37].

Therapeutic interest in the cerebellar nuclei emerged from their extensive connectivity—linking the cerebellum to over three dozen brainstem and spinal targets—and from the accessibility and plasticity of their circuit elements, which may support durable behavioral improvements [29]. Importantly, while Purkinje cells exhibit selective vulnerability across many hereditary and acquired ataxias [38], the cerebellar nuclei are relatively spared, and pathological Purkinje activity is strongly implicated in tremor and ataxic motor disturbances; its suppression ameliorates symptoms in experimental models [39].

Studies employing genetic (e.g., Lhx1/5, pcd5J, Car^8^) and lesion-based ataxia models have therefore increasingly focused on specific cerebellar nuclei—particularly the IN, fastigial nucleus, and DCN as a whole [24–27, 29].

The IN in particular has emerged as a promising target for axial symptoms, including stance and gait incoordination, likely reflecting its close integration with spinocerebellar pathways. Beyond cerebellar cortical influence, the IN receives independent afferents from the magnocellular red nucleus (RNm) [40] and sends reciprocal projections, forming a feedback loop that suggests cerebellar output may be modulated by extracortical signals [37]. Functionally, the IN contributes to locomotor adaptation [41], contains cerebellospinal neurons that engage premotor spinal circuits [42], and supports neuromuscular learning, including modulation of eyelid kinematics and enhancement of reflexive and conditioned responses [43]. Therapeutically, low-frequency DBS of the IN improves mobility and muscle function in ataxic Car^8^ mutant mice, and its combination with skilled motor training further rescues limb coordination [29].

### Targeted Brain Structures: Most Utilized and Promising Sites

Among the various stimulation targets, the DN was the most frequently investigated structure, particularly in hereditary forms such as SCA3 and in acquired cerebellar ataxias, including post-stroke etiologies. This choice is neuroanatomically and pathophysiologically justified by the DN’s central role in the cerebello-thalamo-cortical loop: as the largest and most lateral of the DCN, this nucleus serves as a major output structure of the cerebellum, projecting to the contralateral thalamus and subsequently to the motor and premotor cortices via the dentato-thalamo-cortical pathway. This circuit is critically involved in the planning, timing and execution of voluntary motor activity, all processes that are profoundly disrupted in ataxic syndromes.

A notable technical advancement in DN targeting was described by Diniz et al. (2021) [19], who employed diffusion tensor imaging (DTI) and tractography of the dentato-rubro-thalamic tract (DRTT) to optimize electrode placement. In their approach, high-resolution magnetic resonance imaging (MRI) sequences, including T2-weighted and susceptibility-weighted imaging (SWI), were used to visualize the DN and the trajectory was refined to ensure proximity between the stimulating contacts and the reconstructed DRTT. This methodology reflects an emerging emphasis on the modulation of white matter pathways as potential mediators of clinical efficacy in DBS. Preliminary results demonstrated favorable outcomes in terms of ataxia and tremor without major adverse effects, suggesting that tractography-assisted targeting of the DRTT may enhance the precision and safety of cerebellar stimulation.

Stimulation of the DN aimed to restore cerebellar output and modulate dentato-thalamo-cortical projections, either through open-loop or closed-loop paradigms, which differ substantially in their mechanism and clinical implications.

Several case reports and small studies on DN stimulation have reported modest but measurable improvements in symptoms such as gait, stance and limb coordination. These improvements were often assessed using the Scale for the Assessment and Rating of Ataxia (SARA). In some cases, subjective improvement was also recorded using tools such as the Patient Global Impression of Change (PGIC) [20, 31].

However, the results were not consistent across all patients. Some individuals exhibited only limited or no improvement, and the available studies differed substantially in their design, sample size, ataxia etiology, and stimulation parameters. An additional factor that may account for the heterogeneity of outcomes is that different forms of cerebellar ataxia reflect distinct underlying neuropathological processes [44–46]. Despite these limitations, the DN remains one of the most promising DBS targets for alleviating core symptoms of ataxia, particularly when the DCN are still structurally preserved.

In this regard, an additional consideration concerns the differential responsiveness to DN stimulation observed across hereditary ataxias, particularly in SCA3. Neuropathological studies indicate that Machado–Joseph disease exhibits a pattern of degeneration that, while affecting multiple brainstem and cerebellar structures, tends to spare the DCN more consistently than other dominant ataxias such as SCA1 or SCA2 [47–49]. In SCA3, neuronal loss within the DN is typically moderate and often occurs later in the disease course, whereas in SCA1 and SCA2 the DN undergoes early and severe degeneration. This relative preservation of DN and of the dentato–thalamo–cortical pathway provides a more intact substrate for neuromodulation, potentially enabling DBS to modulate residual cerebellar output more effectively. This mechanistic framework may explain why some SCA3 patients demonstrate clinically meaningful, though variable, improvements in SARA scores following DN-DBS, whereas individuals with more advanced nuclear degeneration show limited or no benefit.

The VIM and VO of the thalamus were common targets in patients with tremor-dominant phenotypes, particularly in FXTAS. Stimulation of these thalamic nuclei achieved consistent tremor reduction, measured using the Fahn-Tolosa-Marin Tremor Rating Scale (FTMTRS) or Tremor Research Group Essential Tremor Rating Assessment Scale (TETRAS). However, these benefits were often counterbalanced by a worsening of gait and speech, particularly in bilateral procedures.

### Heterogeneity Preventing Meta-Analysis

One of the key limitations in this field is the substantial heterogeneity across studies, that exists at multiple levels:

- Patient populations: different forms of ataxia, including hereditary (e.g., SCA, FXTAS) and acquired forms (e.g., stroke, trauma);
- DBS targets: ranging from cerebellar to thalamic and basal ganglia structures;
- Outcomes assessed: some studies focused on tremor, others on gait or dystonia;
- Methodologies: ranging from single case reports to small case series and very few controlled trials.

This heterogeneity limits the generalizability of conclusions and represents a major obstacle to performing a quantitative meta-analysis. Notably, most studies did not explicitly report stimulation parameters such as frequency, amplitude or pulse width, making it difficult to compare outcomes or optimize treatment protocols. Additionally, follow-up durations were highly variable, further hindering the possibility of drawing standardized conclusions.

The variability in stimulation targets, patient types and outcome measures highlights the need for standardized protocols and well-designed randomized controlled trials to clarify the efficacy and optimal application of DBS in ataxia.

Moreover, long-term follow-up data were generally lacking. None of the included studies systematically evaluated the durability of therapeutic effects beyond 24 to 30 months and where follow-up was reported, outcomes varied significantly.

A further limitation is the strong likelihood of publication bias. Because most available evidence consists of isolated case reports or small series, positive or unexpected outcomes are disproportionately represented, whereas unsuccessful or neutral results are rarely published. This selective reporting may overestimate the apparent efficacy of DBS and limits the external validity of current findings, underscoring the need for prospective registries and systematic reporting of all outcomes, including negative ones.

### Lack of Neurophysiological Insight

A critical and recurring gap in the literature is the scarcity of neurophysiological data in humans. This lack of data limits our ability to correlate clinical outcomes with underlying neural activity and to define reliable stimulation biomarkers. It also remains unclear whether the therapeutic effects of DBS in ataxia arise from:

- the restoration or enhancement of physiological cerebellar output, particularly via dentato-thalamo-cortical projections;
- the disruption of pathological oscillatory activity in downstream motor circuits, as proposed in Parkinson’s disease and dystonia;
- the engagement of non-specific neuromodulatory mechanisms, including diffuse network activation or compensatory plastic responses within the central nervous system.

Furthermore, the functional topography of the DN in humans remains poorly characterized, and the specific neural and non-neural elements modulated by DBS are still unknown. This uncertainty hinders the optimization of both target selection and stimulation parameters. Notably, the best clinical improvements with cerebellar targets appear to be associated with low-frequency stimulation, consistent with findings from animal models [28, 29]. In contrast, extra-cerebellar targets tend to yield optimal results with more classical high-frequency stimulation strategies. This contrast is particularly interesting, as it suggests that DBS within cerebellar structures may exert its beneficial effects primarily by facilitating neuronal activity and restoring a more organized cerebellar output, whereas in extra-cerebellar targets the therapeutic benefit may instead stem from the suppression of disorganized ascending signals.

### Comparison With Non-Invasive Neuromodulation Approaches

In recent years, non-invasive neuromodulation techniques such as TMS and tDCS have been investigated as potential therapeutic tools for cerebellar ataxia. Particularly by modulating the dentato–thalamo–cortical pathway and the activities of the prefrontal, parietal, and temporal lobes, the striatum and STN [50], movement disorders such as essential tremor, ataxia, Parkinson’s disease and dystonia can be managed. Meta-analyses report short-term improvements of ~ 1.5–2 points on the SARA or International Cooperative Ataxia Rating Scale (ICARS), with better outcomes in milder cases and repeated sessions [51]. However, these techniques lack the depth and precision to target DCN, limiting their impact on core ataxic features.

In contrast, DBS allows direct, continuous stimulation of structures like the DN, potentially offering more robust and durable symptom control. While DBS is invasive and resource-intensive, it remains the most precise method for modulating cerebellar output, especially in patients with refractory or progressive forms of ataxia. Non-invasive Brain Stimulation may serve as a complementary or preliminary option to assess eligibility for invasive procedures.

Although multimodal strategies combining DBS with other interventions are conceptually appealing, empirical evidence remains scarce. Aside from isolated reports—such as the recent case by Shen et al. [32] describing posterior subthalamic area DBS combined with spinal cord stimulation in SCA12—no systematic data exist on synergistic or additive effects. In clinical practice, DBS is typically integrated with ongoing pharmacological therapy, but studies have not evaluated whether combined approaches outperform DBS alone. Future research should clarify whether multidisciplinary strategies can enhance functional outcomes in cerebellar ataxias.

## Conclusion

This systematic review examined the current evidence on DBS for cerebellar ataxia, a group of rare but clinically impactful disorders that significantly compromise motor coordination, autonomy and quality of life. Although the overall prevalence of ataxia is low, the functional burden on affected individuals is high, often leading to profound disability. In this context, DBS has emerged as a potential symptomatic intervention, particularly for tremor and, to a lesser extent, gait instability and dystonia.

The findings reveal a heterogeneous and evolving landscape regarding the application of DBS for cerebellar ataxia, with variable targets (ranging from cerebellar nuclei to thalamic and basal ganglia structures), diverse patient populations and inconsistent outcome measures. While stimulation of the DN appears most promising based on early clinical reports, several other targets—such as the interposed and fastigial nuclei—have only been explored in preclinical animal models, limiting their immediate translational applicability.

Furthermore, the small number of studies, limited sample sizes and lack of standardized stimulation protocols or long-term follow-up data preclude firm conclusions and prevent quantitative meta-analysis. In addition, although disease-specific and demographic factors such as genetic background, age of onset, disease duration, age at surgery and sex are likely to influence the response to DBS, the current evidence base is too sparse and heterogeneous to allow meaningful comparisons across etiologies. Most reports lack detailed characterization of these variables, and sample sizes are insufficient to identify reliable predictors of outcome, restricting interpretation to very general considerations. Despite these limitations, the available evidence suggests that DBS may offer meaningful functional benefits in carefully selected patients, particularly when cerebellar output pathways remain at least partially preserved.

Given the current lack of effective symptomatic treatments to improve quality of life and functional outcomes in patients with cerebellar ataxia, continued research efforts in this direction are strongly encouraged. Future research should aim to harmonize methodologies, validate outcome measures specific to ataxia and bridge the translational gap from animal models to clinical application.

Furthermore, the small number of studies, limited sample sizes and lack of standardized stimulation protocols or long-term follow-up data preclude firm conclusions and prevent quantitative meta-analysis. In addition, although disease-specific and demographic factors such as genetic background, age of onset, disease duration, age at surgery and sex are likely to influence the response to DBS, the current evidence base is too sparse and heterogeneous to allow meaningful comparisons across etiologies. Most reports lack detailed characterization of these variables, and sample sizes are insufficient to identify reliable predictors of outcome, restricting interpretation to very general considerations. Despite these limitations, the available evidence suggests that DBS may offer meaningful functional benefits in carefully selected patients, particularly when cerebellar output pathways remain at least partially preserved.

Given the current lack of effective symptomatic treatments to improve quality of life and functional outcomes in patients with cerebellar ataxia, continued research efforts in this direction are strongly encouraged. Future research should aim to harmonize methodologies, validate outcome measures specific to ataxia and bridge the translational gap from animal models to clinical application. 

## Data Availability

No datasets were generated or analysed during the current study.

## Abbreviations:

4-AP: 4-Aminopyridine
BFMDRS: Burke–Fahn–Marsden Dystonia Rating Scale
CN: Cerebellar Nuclei
ctDCS: Cerebellar Transcranial Direct Current Stimulation
DBS: Deep Brain Stimulation
DCN: Deep Cerebellar Nuclei
DN: Dentate Nucleus
DRTT: Dentato-Rubro-Thalamic Tract
DTI: Diffusion Tensor Imaging
EEG: Electroencephalography
EMG: Electromyography
FTMTRS: Fahn–Tolosa–Marin Tremor Rating Scale
FXTAS: Fragile X–Associated Tremor/Ataxia Syndrome
GPi: Globus Pallidus Internus
ICARS: International Cooperative Ataxia Rating Scale
IN: Interposed Nucleus
LFPs: Local Field Potentials
MRI: Magnetic Resonance Imaging
NICS: Non-Invasive Cerebellar Stimulation
NOS: Not Otherwise Specified
PGIC: Patient Global Impression of Change
PRISMA: Preferred Reporting Items for Systematic Reviews and Meta-Analyses
PSA: Posterior Subthalamic Area
RCT: Randomized Controlled Trial
RNm: Magnocellular Red Nucleus
RoB: Risk of Bias
SARA: Scale for the Assessment and Rating of Ataxia
SCA: Spinocerebellar Ataxia
STN: Subthalamic Nucleus
SWI: Susceptibility-Weighted Imaging
TETRAS: Tremor Research Group Essential Tremor Rating Assessment Scale
TMS: Transcranial Magnetic Stimulation
tDCS: Transcranial Direct Current Stimulation (non-cerebellar)
VIM: Ventral Intermediate Nucleus of the Thalamus
Vo / VoP: Ventral Oral / Ventral Oral Posterior Nucleus of the Thalamus
ZI: Zona Incerta
